# Effect of Spent Mushroom (*Cordyceps militaris*) on Growth Performance, Immunity, and Intestinal Microflora in Weaning Pigs

**DOI:** 10.3390/ani10122360

**Published:** 2020-12-10

**Authors:** Waewaree Boontiam, Chalong Wachirapakorn, Pheeraphong Phaengphairee, Suchat Wattanachai

**Affiliations:** 1 Department of Animal Science, Faculty of Agriculture, Khon Kaen University, Khon Kaen 40002, Thailand; chal_wch@kku.ac.th (C.W.); perpan@kku.ac.th (P.P.); 2 Department of Surgery and Theriogenology, Faculty of Veterinary Medicine, Khon Kaen University, Khon Kaen 40002, Thailand; suchat@kku.ac.th

**Keywords:** spent mushroom, growth performance, immunity, inflammation, microbial population, weaned pigs

## Abstract

**Simple Summary:**

There are limited published reports regarding the optimal dosage of spent mushroom (*Cordyceps militaris*) as an antibiotic substitute in weaning pigs. This study investigated the effect of various dosages of the spent mushroom in-feed supplement on the growth performance, immunity, inflammation, and microbial count of weaning pigs. The supplementation at a level of 1.5 g/kg spent mushroom improved body weight, average daily gain, average daily feed intake, immunoglobulin A concentration, and *Lactobacillus* spp., whereas it decreased blood cholesterol, interleukin-1, tumor necrosis factor-α, and *Escherichia coli*. This approach provides a basis for the possible use of *C. militaris* spent mushroom as an alternative growth promoter in weaned pigs.

**Abstract:**

There are limited published reports regarding the optimal dosage of spent mushroom. This study investigated the effect of various levels of spent mushroom derived from *C. militaris* as an alternative growth promoter to an in-feed antibiotic on the growth performance, blood profiles, immunoglobulin, inflammation, and microbial count of weaning pigs. A total of 120 pigs (6.63 ± 0.13 kg initial body weight) were blocked by weight and sex in a randomized complete block design. Each treatment had six replicates of four pigs each. The pigs were allotted into five treatments: (1) positive control (PC) with 150 mg/kg colistin; (2) negative control (NC) without antibiotic inclusion; and (3–5) negative control groups with 0.5, 1.0, and 1.5 g/kg of *C. militaris*s pent mushroom (SM), respectively. Blood samples were collected at day 35 for determination of blood-related lipid metabolism and immunity. Fresh fecal samples were collected to examine microbial counts on day 35 postweaning. The results showed that SM at 1.5 g/kg improved the body weight, average daily weight gain, and average daily feed intake of weaning pigs in the overall period (*p* < 0.05). Moreover, the highest dosage of SM caused improvements in the concentrations of high-density lipoprotein, and immunoglobulin A, along with suppressions of total cholesterol, interleukin-1, tumor necrosis factor-α, and *E. coli* (*p* < 0.05). Therefore, the weaned pigs fed a 1.5 g/kg SM diet showed improved growth performance and displayed greater immunoglobulin secretion and lower inflammation, pathogenic population, and cholesterol concentration.

## 1. Introduction

Post-weaning stress is one of the most critical concerns in pig production worldwide. It can contribute to intestinal and immune dysfunctions, which consequently impair growth performance and pig health [1,2,3]. Colistin is a polymyxin antibiotic, extensively used for the control of intestinal infection and diarrhea caused by Gram-negative bacteria in weaning pigs, particularly *E. coli* [4,5]. Even though colistin is effective, the overuse of this antibiotic has increased colistin-resistant *E. coli* prevalence by about 35% in the surviving pigs [6,7]. Therefore, the EU has banned the use of antibiotics as growth-promoting agents in 2003, and the USA followed suit in 2017 [8,9]. A similar regulation has also been approved in many countries, including Brazil, Thailand, and China [10,11,12], in order to prevent multidrug-resistant bacteria, as well as public health concerns, andto offer environmental protection. Thus, in the current research involving swine nutrition, we search for alternatives to antibiotic compounds that would have similar effects as antibiotics in weaning pigs [13,14].

*C. militaris* is well established for its biological functions against diseases and for health protection in humans and animals [15,16]. It is cultivated on a large scale for commercial purposes in many countries [17,18,19]. *C. militaris* production accounts for over 4000 tons per year in China [18] and over 25 million tons worldwide [20]. This increase in mushroom consumption causes the production of greater amounts of undesirable parts of the mushrooms as waste in the environment. Spent mushroom (SM) from *C. militaris* contains various bioactive compounds such as gamma-oryzanol, cordycepin, adenosine, D-mannitol, and polysaccharides [21,22,23]. The use of *C. militaris* significantly improved growth performance and immunity [24,25], in agreement with a previous study in which Nile tilapia were fed 10 g/kg SM [26]. The supplementation of 2 g/kg SM in the diet has recently been reported to stimulate immunoglobulin secretion and antioxidant activity in growing pigs [22]. Activation of the cellular immune response has also been observed in weaning pigs fed with fermented *C. militaris* [25]. However, data concerning in-feed SM supplementation at levels lower than 2 g/kg feed and its use as an antibiotic replacement in weaning pigs have not been published. Our hypothesis was that some active compounds present in SM may activate biological functions in a pig’s body that could positively affect their growth performance and health status. The aim of our research was to increase the value-added SM to the weaned pigs’ diet for sustainable development with the full utilization of mushroom waste for swine production. Consequently, our study evaluated the effect of dietary supplementation of different SM (*C. militaris*) dosages as a colistin replacement on growth performance, immunity, inflammation levels, and intestinal microflora in weaning pigs.

## 2. Materials and Methods

All animal procedures were approved by the Animal Care and Use Committee of Khon Kaen University (Khon Kaen, Thailand) under the official record no. IACUC-KKU50/63.

### 2.1. Animals, Dietary Treatments, and Management

A total of 120 crossbred weaned pigs (Landrace × Large White) × Duroc with an average initial body weight (BW) of 6.63 ± 0.13 kg (28 days old) were used in a 5-week feeding trial. All experimental piglets were randomly allotted into five dietary treatments. Six replicates per treatment and four pigs per pen (two gilts and two barrows) were used in a randomized complete-block design based on their BW and gender. The dietary treatments were (1) PC (positive control, basal diet with 150 mg/kg colistin); (2) NC (negative control, basal diet without colistin supplement); (3) SM05 = NC + 0.5 g/kg SM; (4) SM10 = NC + 1 g/kg SM; and (5) SM15 = NC + 1.5 g/kg SM. The nutrient composition of the spent mushroom is shown in Table 1. The SM powder was included in the diet as a substitute for an equal amount of corn. The SM residue was analyzed for nutrients and some active compounds before in-feed supplementation, as previously reported in our study [22]. A basal diet was prepared in a mash form using a horizontal feed mixer with a capacity of 100 kg for each treatment and blended for 15 min. To avoid contamination between treatments, feed without additive was prepared before feeds supplemented with SM. The latter were based on the basal diet and were made in increasing dosages. The diet was prepared weekly to ensure feed quality (Table 2). All nutrients were formulated to meet or exceed the predicted requirements of NRC [27] during the weaning period and were fed in two feeding programs, Phase I (0 to 14 days postweaning) and II (15 to 35 days postweaning). The diets contained 3392 and 3338 kcal of ME/kg, 22 and 20.90% crude protein, and 1.28 and 1.19% total lysine for Phase I and II, respectively. Feed and water were provided ad libitum via a self-feeder and an automatic waterer throughout the trial.

All piglets were housed in a semi-environmentally controlled building with slatted concrete floors (1.6 m wide × 1.6 m long, stocking density of 0.64 m^2^ for each pig), and rice straw was used as an overnight bedding material during a 2-week period post-weaning to ensure the weaned piglets were kept at the proper temperature, which followed international regulations (the EU Directive 2010/63/EC for animal experiments). The housing temperature was controlled using a mechanical ventilation system when the temperature was above 30 °C during the first week and was gradually decreased by 1 °C each week of the trial. The pigs were free from Aujeszky’s disease, atrophic rhinitis, transmissible gastroenteritis, porcine reproductive and respiratory syndrome and Salmonellosis, thus vaccinations were not administered during the experimental period.

### 2.2. Growth Performance

Individual pigs’ BW and feed intake were measured for each pen at 0, 14, and 35 days to calculate their average daily gain (ADG), average daily feed intake (ADFI), and gain-to-feed ratio (G:F). Mortality was recorded daily to adjust the feed intake in each pen.

### 2.3. Diarrhoea Occurrence

The number of diarrheic piglets in each pen was visually assessed every morning by two observers from Day 1 to 10 during the feeding period. Faecal consistency was scored as follows: (0), hard faeces (normal); (1), slightly soft faeces; (2), semiliquid faeces; and (3), watery faeces. The pigs with daily faecal consistency over 2 were identified as having diarrhea. The diarrhoeal rate was calculated using the following equation: the number of diarrhoeal pig/(the total number of pigs × experiment day) × 100 [13].

### 2.4. Blood Profiles

On Day 35, blood samples were randomly collected from 30 pigs (six pigs per treatment; three gilts and three barrows) after overnight fasting via jugular venipuncture using sterilized syringes with needles. This procedure was performed within a minute to diminish handling stress. The representative samples (8 mL) were immediately transferred into non-anticoagulant vacuum tubes (Becton Dickinson Vacutainer Systems, Franklin Lakes, NJ, USA). Then, the samples were stored at room temperature for 2 h and subsequently centrifuged at 3000× *g* at 4 °C for 10 min to obtain serum. The samples were frozen at −20 °C and further used for determination of metabolic profiles, immunoglobulins, and inflammation.

The metabolic profiles of aspartate aminotransferase (AST, Boehringer Mannheim, Germany), triglyceride (TG), total cholesterol (TC), high-density lipoprotein (HDL), and low-density lipoprotein (LDL) were quantified using enzymatic colorimetric methods with a commercial kit (Zhongsheng Biochemical, Beijing, China) following the manufacturer’s instructions. Briefly, plasma was diluted by a 10-fold dilution with phosphate buffer saline (pH 7.0–7.2) for determining blood profiles. Each microplate received 100 µL of conjugated antibody specific, incubated at 37 °C for one hour, which was subsequently auto washed three times. Then, 50 µL of sulfuric acid solution was added as a stop solution. The concentration of AST was quantified by an automatic analyser at a wavelength of 450 nm. The concentrations of metabolic profiles were prepared by diluting plasma samples at 1:200 with assay diluent and placed to the 96-well microliter plate at 50 samples of 100 µL each. Then, 50 µL of each reaction reagent was added to each well and mixed to homogenise. All of the samples were incubated at room temperature for 45 min before an assay was run at an absorbance of 530 nm using a microplate reader. Each sample was immediately performed in duplicate.

### 2.5. Immunity

The concentrations of immunoglobulins (IgA, IgG; porcine immunoglobulin kit, Bethyl Laboratories, Inc., Montgomery, AL, USA), interleukine-1β (IL-1β, R&D System, Minneapolis, MN, USA), and tumor necrosis factor alpha (TNF-α, R&D System Inc., Minneapolis, MN, USA) were quantified by ELISA methods using commercial reagent kits. After thawing, samples were diluted with phosphate-buffered saline to 10- and 100-thousand-fold dilutions, respectively. The diluted porcine IgA samples were added to each plate and incubated at 37 °C for 3 h. Then, 100 µL of bionylated procine IgA, enzyme-conjugated liquid antibody, and colour reagent liquid were placed in each sampling plate and subsequently incubated at 37 °C for one hour. The HRP-conjugate reaction (100 µL for each) was used to determine the IgG concentration. The samples were washed five times using an automatic plate washing after incubation at 37 °C for one hour. Then, the chromogen solution was added, and the samples were placed in room temperature for 15 min prior to the stop solution. Each sampling plate (96-well plate) was subsequently measured for immunoglobulin quantity using an automatic microplate reader at an absorbance of 450 nm. Each sample was run in duplicate to avoid variations.

To quantify IL-1β, 100 μL of serum was placed in each well, incubated for 30 min at room temperature, and gently washed five times. Detection reagents A and B (100 µL of each) were placed in each plate, covered with a plate sealer, and incubated for 60 and 30 min at 37 °C, respectively. Each sampling plate was aspirated of liquid and washed five times before 90 µL of TMB substrate was added. The samples were incubated for 20 min at 37 °C. Then, 90 µL of stop solution was added in the same order as the TMB substrate solution until optimum colour change. The TNFα concentration was measured with 50 µL of serum. The samples were placed on a sampling microplate reader and coated with biotinylated antibody reagent. To detect substrate, 3,3′,5,5′-tetramethylbenzidine was used, and the stop solution contained 2 mol/L H_2_SO_4_. The absorbance was determined immediately at 450 nm in accordance with the manufacturer’s guidelines. All representative samples were analyzed in duplicate, with six samples per treatment (*n* = 60).

### 2.6. Intestinal Microflora

At the end of the experiment, one pig from each replication (six pigs per treatment with three gilts and three barrows, *n* = 30) was randomly selected for faecal collection via rectal dilation with a plastic container (50-mL conical centrifuge tube). After faecal collection, the tube was covered in a sealed plastic bag, packed in a polystyrene box containing freezer packs, and directly delivered to the laboratory to determine the microbial population on the following day. Approximately 1 g of fecal sample was diluted with 0.9% sterile saline solution and blended by vortex mixing. The viable counts of each microbial colony were performed in serial 10-fold dilutions (10^−1^–10^−8^). *Salmonella-Shigella* agar, MacConkey agar, and Rogosa and Sharpe agar were used to enumerate *Salmonella* spp., *E. coli*, and *Lactobacillus* spp., respectively. Sixty grams of each agar was prepared by suspending the agar in one litre of deionised water and gently mixing it for one minute. The agars were subsequently autoclaved at 118 °C for 15 min and then poured into plates. The representative samples of faecal dilutions were overlaid on the appropriate selected agar plates and subsequently incubated for 48 h at 37 °C in aerobic conditions. The viable spots of each colony were counted immediately upon detecting each bacterial growth after 24 h of incubation. The presence of each bacterium is presented as the logarithm of colony-forming units (cfu)/g. All representative samples were detected twice.

### 2.7. Statistical Analysis

Data were analyzed in a randomized complete-block design using the GLM procedure of the SAS software package (SAS University Edition, version 3.8, SAS Institute Inc., SAS Drive, Cary, NC, USA). Each pen (six replicates) was an experimental unit for growth performance measurement, whereas each individual weaned pig (six pigs per treatment, *n* = 30) was an experimental unit for metabolic profiles, immunity, inflammation, and microbial proliferation. Significant differences among treatments were defined by Duncan’s new multiple range test. The results are expressed as the mean and standard error of the mean. The alpha level used for determining significance was 0.05.

## 3. Results

### 3.1. Growth Performance

As shown in Table 3, pigs fed SM15 had greater final BW (*p* = 0.005) and ADG (*p* = 0.007) for the overall period than did those fed the NC diet. The significant differences in BW, ADG, and ADFI in the SM15 treatment were comparable to those in the PC treatment. In addition, the ADFI was significantly increased in the SM15 treatment compared to the NC treatment from Days 15 to 35 (*p* = 0.004) and the overall period (*p* = 0.003). The BW, ADG, and ADFI were higher for pigs fed SM05 treatment compared with the NC pigs in the overall period (*p* < 0.05). However, the dietary treatments did not affect ADG, and G:F ratio from 0 to 14 days or from 15 to 35 days. Regarding the animals’ health status throughout the experimental period in all groups, the pig mortality did not occur (data not shown). The percentage of diarrhoea in the SM10 was lower than that in the NC treatment (3.17 vs. 4.54), but there was no significant difference between the two treatments (*p* = 0.402).

### 3.2. Blood Profiles

As shown in Table 4, feeding weaned pigs a SM15-supplemented diet significantly decreased TC (*p* = 0.044), whereas HDL (*p* = 0.039) concentrations were increased compared to those in the PC and NC treatments after the 5-week growth trial. However, the concentrations of AST, TG, and LDL were unaffected by the antibiotic and SM supplement treatments.

### 3.3. Immunity

As shown in Table 5, the IgA concentration was higher in the SM15 treatment compared to the PC and NC treatments (*p* = 0.024). Furthermore, lower secretions of inflammatory cytokine IL-1β (*p* = 0.004) and TNF-α (*p* = 0.001) were observed in the SM15-supplemented diet compared to the controls. The supplementation of SM05 and SM10 had positive effects on lower IL-1β and TNF-α secretions compared to the NC treatment (*p* < 0.05). The pigs fed the NC diet showed increased IL-1β secretion compared to those that consumed the PC treatment (*p* = 0.004); however, no significant difference was observed between the PC and NC in terms of TNF-α secretion. No significant difference was observed for IgG concentration among dietary treatments.

### 3.4. Intestinal Microflora

As shown in Table 6, the PC, SM10, and SM15 diets significantly decreased the population of *E. coli* (*p* = 0.041) in comparison to the NC treatment. Furthermore, a higher abundance of *Lactobacillus* spp. was observed in the SM05 diet than in the control diets (*p* = 0.043). No significant difference was observed in the microbial count of *Salmonella* spp. among the dietary treatments.

## 4. Discussion

Notably, the inclusion of SM improved the growth performance of weaning pigs. This improvement agrees with previous observations showing that the addition of 1, 2, and 10 g/kg SM significantly improved the zootechnical performance of broiler chickens, growing pigs, and Nile tilapia, respectively [23,24,26]. This positive effect may be caused by the weaning pigs being able to uptake nutrients more efficiently and maintain their health status, resulting in better growth performance. This is in agreement with the immunity and faecal microbial count findings; however, future research is needed to investigate the effects of SM on nutrient digestibility in weaning pigs. This result suggests that the combination of major active compounds in SM may have a modulating effect to enhance growth performance similar to the PC diet, and may thus be considered to replace antibiotic use during the weaning period. However, in this study, the SM treatments did not have an impact on the diarrhoea occurrence. It might be influenced by a short duration of data collection.

Changes in blood profiles can determine health status in animal bodies [25,28]. The current study demonstrated that the AST concentration was unaffected by dietary treatments. This implies that the inclusion of *C. militaris* SM is nontoxic to weaned pigs. Furthermore, previous studies showed that weaned pigs are highly susceptible to hepatic damage with higher AST production when fed a diet without *C. militaris* waste supplement [25]. In terms of the hypercholesteromic profile, the inclusion of 1.5 g/kg SM resulted in a cholesterol concentration lower than those in the control groups. The lower production of total cholesterol positively induced by cordycepin through the inhibition of glycerol-3-phosphate acyltransferase and HMG-CoA reductase has been established in prior studies [29,30]. It is well documented that HMG-CoA reductase is a key regulatory enzyme of cholesterol synthesis [29]. This finding was consistent with a previous study showing that the inclusion of 25 and 50 mg/kg cordycepin significantly decreased the levels of TC, TG, and LDL in hamsters and rats [31]. Another possible effect would be due to the presence of γ-oryzanol in SM residue, which plays a role to some extent, by either increasing cholesterol absorption or enhancing cholesterol conversion [32]. The mechanism of lowering cholesterol is activated by cholesterol acyl transferase carrying cholesterol from the peripheral tissues to the liver [33,34]. Consequently, this converts cholesterols and lipid fractions from blood circulation into faecal bile acids for further excretion [35]. Furthermore, the SM contained dietary fibre (163 g kg^−1^), which could induce a hypercholesterolemic effect through increased production of short-chain fatty acids, which in turn can suppress hepatic and intestinal cholesterol synthesis and decrease cholesterol levels [36,37]. However, this is in contrast to previous findings that did not observe changes in the cholesterol level in pigs consuming an SM diet [23,25]. The controversial outcome may have been caused by the pig growth stage, diet composition, animal health status, or environmental variation. We also found that pigs fed an SM-supplemented diet had elevated HDL cholesterol. Generally, an increase in HDL reflects the capacity to activate cholesterol efflux into the hepatocytes for further metabolites. These positive effects can be used in-feed supplements to control the lipid metabolism of weaning pigs.

Immunoglobulin A and IgG play an important role in humoral immunity among animals. However, the production of immunoglobulins is reduced during the weaning period of pig growth [38]. The production of acute-phase protein also increases rapidly, especially haptoglobin in the serum [39], which subsequently impaired the intestinal barrier function and growth performance of pigs [40]. Our findings show improved IgA concentrations in weaning pigs that consumed the SM15-supplemented treatment. The present outcome, explained by SM supplementation, could be influenced by higher amounts of mannanoligosaccharides (MOS). Previous study has shown the positive effects of a MOS-supplemented diet on the secretory enhancement of IgA [41]. This is attributable to the modulation of local immune response via the mannose-binding receptors located in the surface area of the mucosal cells [42]. According to Davies et al. [43], the supplementation of phosphorylated mannan (2 g/kg diet) altered the T lymphocytes of the weaning pigs after 21 days of feeding [43]. Our finding also observed that the secretions of these acute-phase proteins were lowered by the SM supplement treatments. One possible explanation is that the SM from *C. militaris* plays an important role in modulating macrophage function through the destruction of pathogenic microbes [25,44]. This activation subsequently increases oxygen radicals, which have effective antimicrobial activity. These agents can synthesize antimicrobial peptides and protease to ingest extracellular parasites and block proinflammatory signals [40]. The present study also found that the SM of *C. militaris* with the jasmine rice formula contained 48.32 µg/100 g dry weight of gamma-oryzanol [23], which has potential effects on the immune system that may protect against bacterial invasion [45]. It might be possible for the SM to change the barrier function of the intestinal mucosa, therefore inhibiting inflammatory action to pathogenic invasion, which is in accordance with lower *E. coli*. Therefore, the increase of IgA and the decrease of inflammatory cytokines is an effective approach to recovering the immune status of weaned pigs.

Intestinal microflora plays an important role in animal health and productivity [46]. Differences in the microbial population following SM supplementation are expected to benefit the overall health and productivity of weaned pigs. The effectiveness of SM in the reduced populations of pathogenic Gram-negative *E. coli* and increased beneficial *Lactobacillus* spp. could be due to the enrichment of polysaccharides [47]. Galactomanna is a main source of polysaccharide in *C. militaris* and is useful for prebiotic production of (MOS). In vitro studies have shown that enteric pathogens are more likely to attach to the intestinal lumen than to the epithelium, which further reduces their colonization [48,49]. This reduction in the number of pathogenic bacteria increases the health-promoting bacteria in the host’s gastrointestinal tract [50,51]. This supports the faecal abundance of *Lactobacillus* spp., whereas a reduction of *E. coli* was found in the current study, in agreement with previous studies. The enhancement of these bacteria consequently produces bacteriocin and enterocin, which activate the competition and proliferation of beneficial flora, whereas they inhibit pathogenic bacteria [52,53]. Thus, SM may use to control diarrhea as it has higher potential to decrease fecal *E. coli* in weaning pigs than the NC diet alone.

## 5. Conclusions

Supplementation with 1.5 g/kg *C. militaris* SM resulted in greater BW, ADG, and ADFI in weaned pigs, which appeared to be comparable to the effects of a colistin diet during two feeding periods. Furthermore, the inclusion of SM modulated HDL, IgA, and *Lactobacillus* spp., whereas it inhibited TC, IL-1β, TNFα, and *E. coli* without hepatic alterations. This approach may be considered as an alternative to reduce colistin use in the diet of weaning pigs.

## Figures and Tables

**Table 1:** Nutrient composition of spent mushroom (*C. militaris*).

Items	Amount (% As-Fed Basis)
Dry matter	93.62
Crude protein	7.82
Crude fiber	16.34
Ether extract	3.67
Ash	4.48

**Table 2:** Ingredients and chemical composition of basal diet (% as fed basis).

Ingredient	Phase I (0–14 Day)	Phase II (15–35 Day)
Broken rice	35.16	28.16
Maize (8.4% CP ^1^)	20.00	34.63
Soybean meal (45.6% CP)	3.61	16.60
Full-fat soybean	27.83	13.21
Fish meal (58% CP)	5.00	5.00
Skimmed milk	6.00	-
Dicalcium phosphate	1.70	1.70
Sodium chloride	0.35	0.35
Vitamin-mineral premix ^2^	0.35	0.35
Calculated values (%)		
Metabolisable energy (kcal/kg)	3485	3330
Crude protein	22.00	20.90
Calcium	0.92	0.84
Available phosphorus	0.83	0.68
Lysine	1.28	1.19
Methionine + cystine	0.58	0.53
Tryptophan	0.25	0.23
Fibre	1.79	2.58
Analysed composition (%)		
Metabolisable energy (kcal/kg)	3392	3338
Crude protein	22.03	20.84
Ether extract	7.23	6.54

^1^ CP = crude protein. ^2^ Premix contents (per kg): retinol 8,400 IU; vitamin D_3_, 945 IU; vitamin E, 0.0126 g; vitamin K, 0.0021 g; thiamine, 0.0011 g; riboflavin, 0.0022 g; pyridoxine, 0.0016 g; nicotinic acid, 0.0126 g; biotin, 0.0315 mg; cyanocobalamin, 0.02 mg; folic acid, 0.0053 g; choline, 0.175g; pantothenic acid, 0.063 g; CuSO_4_, 0.126 g; FeSO_4_, 0.105 g; manganese, 0.021 g; cobalt, 0.0007 g; iodine, 0.0007 g; Na_2_SeO_3_, 0.00007 g.

**Table 3:** Effect of spent mushroom (*C. militaris*) on growth performance and diarrhoea occurrence in weaning pig ^1,2^.

Items	PC	NC	SM05	SM10	SM15	SEM ^3^	*p*-Value
BW (kg)							
Initial	6.81	6.59	6.60	6.54	6.63	0.130	0.627
14day	10.93	9.89	10.43	10.67	10.66	0.299	0.189
35 day	19.86 ^a^	17.08 ^c^	18.39 ^b^	18.88 ^a,b^	19.89 ^a^	0.412	0.005
ADG (g)							
0–14 day	293.81	235.95	273.57	295.36	287.98	24.774	0.434
15–35day	425.24	342.22	379.21	390.95	439.53	24.755	0.082
Overall	372.67 ^a,b^	299.72 ^c^	336.95 ^b^	352.71 ^a,b^	378.91 ^a^	11.560	0.007
ADFI (g)							
0–14 day	387	363	378	381	377	9.372	0.483
15–35 day	767 ^a^	669 ^c^	728 ^a,b^	697 ^b,c^	751 ^a^	13.706	0.004
Overall	615 ^a^	546 ^c^	588 ^a,b^	571 ^b,c^	602 ^a^	8.938	0.003
G:F ratio							
0–14 day	0.764	0.651	0.734	0.776	0.768	0.073	0.734
15–35 day	0.554	0.513	0.521	0.561	0.589	0.036	0.577
Overall	0.606	0.550	0.574	0.618	0.632	0.023	0.120
Diarrhea rate (%)	3.82	4.54	3.71	3.17	3.70	0.458	0.402

^1^ BW = body weight, ADG = average daily gain, ADFI = average daily feed intake, G:F = gain: feed ratio, PC = positive control with 150 mg/kg diet of colistin, NC = negative control without colistin or SM supplementation; SM05 = NC + 0.5 g/kg SM; SM10 = NC + 1 g/kg SM; and SM15 = NC + 1.5 g/kg SM. ^2^ Mean values represent six replicates per treatment. ^3^ Standard error of the mean (*n* = 30). ^a–c^ Mean values with uncommon superscripts represent statistically significant differences (*p* < 0.05).

**Table 4:** Effect of spent mushroom (*C. militaris*) on metabolic profiles in weaning pigs ^1^.

Items	PC	NC	SM05	SM10	SM15	SEM ^2^	*p*-Value
AST (U/L)	56.16	58.69	59.97	61.76	52.55	5.500	0.789
TG (mg/dL)	52.69	47.62	53.71	46.67	54.28	6.230	0.854
TC (mg/dL)	73.86 ^a^	70.16 ^a^	59.11 ^a,b^	61.71 ^a,b^	47.68 ^b^	5.941	0.044
HDL (mg/dL)	28.74 ^b^	24.13 ^b^	36.16 ^a,b^	34.12 ^a,b^	45.44 ^a^	4.593	0.039
LDL (mg/dL)	32.38	23.58	25.85	31.83	33.62	4.012	0.331

^1^ AST = aspartate aminotransferase, TG = triglyceride, TC = total cholesterol, HDL = high density lipoproteins; LDL = low density lipoprotein; PC = positive control with 150 mg/kg diet of colistin, NC = negative control without colistin or SM supplementation; SM05 = NC + 0.5 g/kg SM; SM10 = NC + 1 g/kg SM; and SM15 = NC + 1.5 g/kg SM. ^2^ Standard error of the mean (*n* = 30, six samples per treatment). ^a,b^ Mean values with uncommon superscripts represent statistically significant differences (*p* < 0.05).

**Table 5:** Effect of spent mushroom (*C. militaris*) on immunity and inflammatory responses in weaning pigs ^1^.

Items	PC	NC	SM05	SM10	SM15	SEM ^2^	*p*-Value
IgA (mg/mL)	0.71 ^b,c^	0.57 ^c^	0.78 ^a,b,c^	0.82 ^a,b^	0.96 ^a^	0.075	0.024
IgG (mg/mL)	0.59	0.57	0.69	0.72	0.76	0.081	0.393
IL-1β (pg/mL)	77.16 ^b^	95.52 ^a^	70.87 ^b,c^	64.78 ^b,c^	59.24 ^c^	4.830	0.004
TNF-α (pg/mL)	3011.52 ^a,b^	3227.58 ^a^	2817.70 ^b,c^	2751.72 ^c^	2524.06 ^d^	74.174	0.001

^1^ IgA = immunoglobulin A, IgG = immunoglobulin G, IL1 = interleukin-1β (IL-1β), and TNFα = tumor necrosis factor-α; PC = positive control with 150 mg/kg diet of colistin, NC = negative control without colistin or SM supplementation; SM05 = NC + 0.5 g/kg SM; SM10 = NC + 1 g/kg SM; and SM15 = NC + 1.5 g/kg SM. ^2^ Standard error of the mean; *n* = 30 (six samples per treatment). ^a–d^ Mean values with uncommon superscripts represent statistically significant differences (*p* < 0.05).

**Table 6:** Effect of spent mushroom (*C. militaris*) on microbial proliferation in weaning pigs ^1^.

Items	PC	NC	SM05	SM10	SM15	SEM ^2^	*p*-Value
Fecal microbial counts, log_10_ cfu/g							
*Salmonella* spp.	4.93	6.63	5.16	5.05	5.32	0.425	0.064
*Escherichia coli*	4.17 ^b^	6.08 ^a^	4.75 ^a,b^	3.66 ^b^	4.37 ^b^	0.524	0.041
*Lactobacillus* spp.	6.08 ^b^	6.41 ^b^	8.97 ^a^	8.24 ^a,b^	8.38 ^a,b^	0.744	0.043

^1^ PC = positive control with 150 mg/kg diet of colistin, NC = negative control without colistin or SM supplementation; SM05 = NC + 0.5 g/kg SM; SM10 = NC + 1 g/kg SM; and SM15 = NC + 1.5 g/kg SM. ^2^ Standard error of the mean; *n* = 30 (six samples per treatment). ^a,b^ Mean values with uncommon superscripts represent statistically significant differences (*p* < 0.05).
